# Triptan Use and Potential Undertreatment in Migraine: A Retrospective Cohort Study

**DOI:** 10.3390/jcm15041437

**Published:** 2026-02-12

**Authors:** Yael Barer, Moshe Hoshen, Sivan Gazit, Sarah Sharman Moser, Matanya Tirosh, Danna Davivdovici, Erel Domany, Amnon Mosek

**Affiliations:** 1 Maccabi Institute for Research and Innovation, Maccabi Healthcare Services, Tel Aviv 6812509, Israel; 2 Pfizer Pharmaceuticals Israel LTD, Herzliya 4672509, Israel; 3 Department of Neurology and Headache Clinic, Rambam Medical Center, Haifa 3109601, Israel; 4 Headache and Facial Pain Clinic, Hod Hasharon 454529248, Israel

**Keywords:** migraine, triptans, persistence, discontinuation, naïve users

## Abstract

**Background/Objectives**: While triptans remain the standard of care for moderate-to-severe migraine, the high discontinuation rates and remarkably low prescribing rates of triptans reflect suboptimal therapeutic optimization. **Methods**: This retrospective cohort study utilized data from Maccabi Healthcare Services (MHS), the second-largest healthcare provider in Israel, spanning 10 years (2012–2022). We identified naïve triptan users aged 21 years or older and evaluated triptan initiation and discontinuation patterns. Median time to discontinuation was assessed using Kaplan-Meier curves. **Results**: Of 2.8 million MHS members, 91,619 (3.4%) individuals had at least one triptan dispensation or prescription during the study period, including 41,297 triptan-naïve patients who met the study criteria. The median age at triptan initiation was 38.4 years (interquartile range: 28.2–48.0), and 75.6% were female. Overall, the median time to discontinuation was 7.7 months (95% confidence interval: 7.1–8.4). Discontinuation occurred in 70% of the cohort, with approximately 74% of triptan users purchasing only a single triptan formulation throughout the study period. Subgroup analyses by age, sex, socioeconomic status, and anxiety/depression prevalence demonstrated consistent discontinuation patterns across demographic and clinical characteristics, with median time to discontinuation remaining under 12 months in nearly all subgroups. Annual median time to discontinuation consistently remained under 12 months throughout the study period while showing a significant decreasing trend. **Conclusions**: The low rates of triptan use and high discontinuation rates suggest a possible undertreatment of migraine in Israel. These results highlight the need for improved patient and physician education, and enhanced follow-up protocols.

## 1. Introduction

Migraine is a prevalent and debilitating neurological disorder characterized by recurrent severe headache attacks, often accompanied by nausea, vomiting, and hypersensitivity to light and sound [1]. Clinical diagnosis is standardized by the International Classification of Headache Disorders (ICHD-3) criteria, which distinguish migraine based on attack duration, pain characteristics, and associated symptoms [2]. The global burden of migraine is substantial, affecting approximately one in seven individuals worldwide and significantly impacting quality of life and productivity [1,3]. Both direct and indirect healthcare costs contribute to migraine’s considerable economic impact [4].

Migraine management encompasses two primary approaches: acute treatment to reduce the severity and duration of individual attacks, and preventive treatment to decrease attack frequency [5,6]. Preventive strategies typically involve daily pharmacological agents—such as beta-blockers, topiramate, or CGRP-targeted therapies—and are indicated for patients experiencing frequent or debilitating attacks [7]. In refractory cases, interventional procedures like nerve blocks or sphenopalatine ganglion (SPG) blocks may also be utilized to modulate pain pathways and provide symptomatic relief [8]. Over-the-counter analgesics and prescribed triptans are the most commonly used medications for acute treatment. Despite the longstanding use of triptans as the standard of care for moderate-to-severe migraine for over two decades [5,6,9], triptan discontinuation rates remain high globally, showing consistent patterns across diverse geographic and economic regions. In the United States, 50–65% of new users fail to refill their initial prescription within 12–24 months, with 30–60% discontinuing within the first year [10,11,12,13]. Comparable trends are observed in Europe; in the UK, France, and Germany, over 55% of patients never refill their index prescription, with persistence dropping below 14% at two years [14,15]. This pattern extends to Asian markets; for instance, in Taiwan, only 34.3% of users refilled their initial prescription, with a 4% retention rate at two years [16]. In developing regions such as Pakistan, where triptan prescription rates may be lower (38%), the median time to discontinuation is approximately 20 months [17]. Of triptan initiators, 90% use only one type of triptans, 8% use two different types of triptans and only 1% use three or more [11]. Overall, there is a great similarity in triptan discontinuation rates. This may reflect a universal challenge in migraine management, with patient-related factors outweighing differences in healthcare systems or drug availability. Guidelines recommend trying multiple triptans due to heterogeneity in patient response, as individuals may respond differently to various agents within this drug class. While discontinuation may result from migraine remission, it is more likely stems from insufficient efficacy, poor tolerability, or adverse effects [18]. Patients who discontinue triptan treatment face higher economic burdens, with costs escalating for those who discontinue multiple triptan formulations [19].

While triptan discontinuation has been documented in various global settings including Israel [20,21,22,23], there remains a lack of large-scale, long-term evidence that captures the nuances of the “patient journey” over an extended period. Most existing studies in Israel have been limited by small cohorts or short follow-up durations, leaving a gap in our understanding of long-term persistence and switching behaviors within a universal healthcare framework. Therefore, this population-based retrospective study leverages a 10-year database of 2.8 million individuals to evaluate real-world triptan treatment patterns. We sought to specifically address research questions regarding the long-term rates of persistence, the frequency of switching between formulations, and the time to discontinuation. We hypothesized that even in a large, stable healthcare system, triptan discontinuation rates would be high and persistence duration would be short, highlighting a significant and ongoing unmet need in acute migraine management.

## 2. Materials and Methods

### 2.1. Study Design and Settings

This retrospective cohort study utilized de-identified data from Maccabi Healthcare Services (MHS), the second-largest health insurer-provider in Israel, covering 28% of the population with an annual migration rate of less than 1%. Data are automatically collected and include comprehensive laboratory data from a single central lab, full pharmacy prescription and dispensation data, and extensive demographic data on each member. MHS uses the International Classification of Diseases, Ninth Revision, Clinical Modification (ICD-9-CM) coding systems, as well as self-developed coding systems to provide more granular diagnostic information beyond the ICD codes. Medications are coded according to the Israeli coding system with translations to Anatomical Therapeutic Chemical (ATC) coding system wherever available. Procedures are coded using Current Procedural Terminology (CPT) codes. This study was conducted and reported in accordance with the Strengthening the Reporting of Observational Studies in Epidemiology (STROBE) guidelines.

### 2.2. Study Period and Eligibility

Within the MHS database (2000–2023) we identified all MHS members aged 21 years or older (to prevent selection and ethnicity bias, as some ethnic populations between 18 and 21 are generally enlisted in the military and therefore not enrolled in the health plan) who had at least one dispensation of triptans (e.g., Eletriptan, Naratriptan, Rizatriptan, Sumatriptan, Zolmitriptan). Patients’ migraine diagnoses were not captured due to low validity. Patients were included if their first purchase occurred between 2012 and 2022. The index date was defined as the earlier of the first triptan prescription or dispensation. Eligible patients had an index date between 2012 and 2022 and had been enrolled in the health plan for at least one year before (to assure incident triptan users and in order to capture accurate baseline characteristics) and after the index date (to allow sufficient time of follow-up to observe treatment patterns). The end of follow-up was defined as the earliest of the following: (1) date of death, (2) end of enrollment in MHS, or (3) 31 December 2023, the end of the study period.

### 2.3. Discontinuation Definition

Since triptans are typically used on an as-needed basis (pro re nata [PRN]), accurately determining the exact duration of medication use from pharmacy dispensations or prescriptions is challenging. Specifically, the final observed dispensation for a PRN medication does not specify a defined use period. For this study, the triptan persistence time was defined as the interval from the index date to the last observed dispensation, plus an additional “use period” of 90 days following the last dispensation [16]. A sensitivity analysis was conducted by applying a last observation carried forward (LOCF) approach, which carried forward the number of gap days between the last refill and the preceding fill. Discontinuation was defined as the absence of additional dispensations between the end of the triptan exposure period and the end of the follow-up period, except where treatment cessation was due to contraindications (e.g., ischemic bowel disease, Selective Serotonin Reuptake Inhibitor [SSRI] initiation, Monoamine oxidase inhibitor [MAOI], liver failure, ischemic heart disease [IHD], ischemic stroke and/or transient ischemic attack [TIA], vascular dementia, or pregnancy); in this case, the triptan exposure period was censored.

### 2.4. Patients’ Characteristics

Demographic characteristics of patients were evaluated, including age at the index date, sex, socioeconomic status (SES) [24], socio-religious affiliation (Arab descent, Ultra-orthodox Jew, or general Jewish population), and smoking status (ever/never). Comorbidities [25] assessed prior to the index date (ever and one year-prior) included anxiety and depression, irritable bowel syndrome (IBS), cervical pain, fibromyalgia, fatigue, insomnia, rheumatic diseases (including Osteoarthritis, Rheumatoid arthritis, Lupus, and Gout) and overactive bladder syndrome (OAB) based on at least one diagnosis during the baseline period. The use of contraceptive and hormone replacement therapies (HRT) was also examined. Additionally, the specialty of the first prescribing physician (general practitioner [GP], neurologist, pain specialist, or other) and number of visits to any health provider during the year prior index date are presented.

### 2.5. Statistical Methods

Descriptive statistics are reported as counts (n) and percentages (%) for categorical variables, and as mean (±Standard Deviation [SD]), median, quartiles (interquartile range [IQR]), minimum, and maximum for continuous variables, as appropriate. Time to discontinuation was analyzed using Kaplan-Meier survival curves and compared across groups using the log-rank test. This approach was selected to provide a transparent, real-world representation of triptan utilization patterns. Sensitivity analyses included: (1) defining discontinuation using the LOCF method, and (2) defining triptan user as individual with more than one dispensation. Several subgroups’ analyses were performed: (1) by sex, (2) age at initiation, (3) SES, (4) first prescribing physician, (5) number of visits to any healthcare provider in the year prior treatment initiation, and (6) prevalence of depression and/or anxiety diagnosis during the year prior to treatment initiation. Analyses were conducted using R statistical software version 4.5.1 (R Foundation for Statistical Computing, Vienna, Austria).

### 2.6. Bias Mitigation and Confounding

To mitigate potential biases inherent in retrospective designs, several steps were taken. Selection bias was addressed by excluding the 18–21 age group to prevent demographic skewing related to military service. To minimize misclassification of discontinuation in PRN usage, we utilized a 90-day grace period and conducted sensitivity analyses (LOCF). Furthermore, to address confounding by medical necessity, patients with incident contraindications to triptans during follow-up were censored at the time of the contraindication diagnosis.

## 3. Results

**Figure 1:**
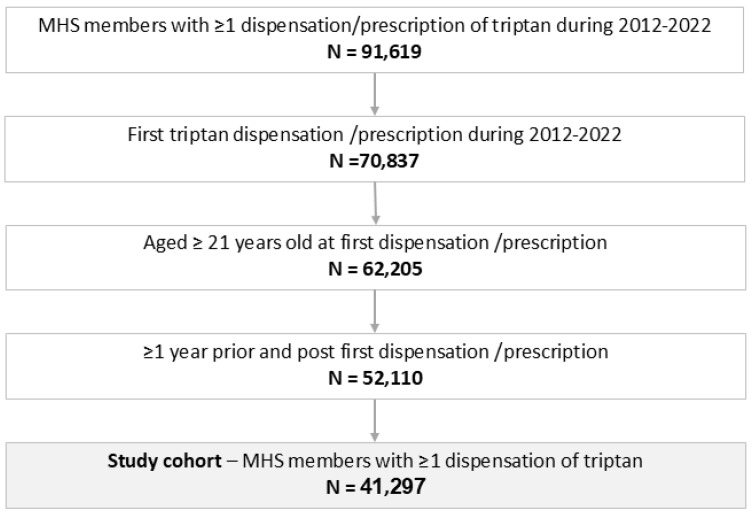
Flow diagram of study population selection. Flow chart of selection of adults initiating triptan treatment, 2012–2022. MHS: Maccabi Healthcare Services.

**Figure 2:**
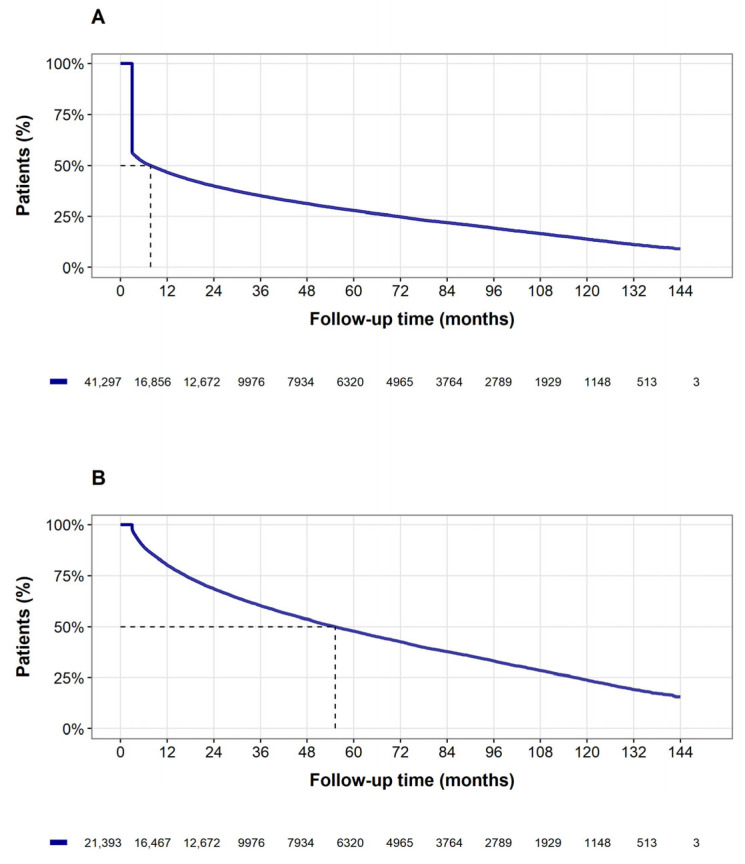
Kaplan-Meier curves of triptan treatment discontinuation. Time to treatment discontinuation (in months) from triptan initiation among (**A**) all individuals with at least one triptan dispensation and (**B**) individuals with more than one triptan dispensation. Numbers below curves represent individuals at risk for discontinuation at each time point.

Of 2.8 million MHS members, 91,619 individuals had at least one triptan dispensation or prescription during the study period, and 41,297 met the study criteria, (Figure 1) with median age at triptan initiation (index date) of 38.4 years (IQR: 28.2, 48.0), and 75.6% females (Table 1). Overall, median time to triptan discontinuation was 7.7 months (95% confidence interval [CI]: 7.1–8.4 months, Figure 2A) with a similar result in the LOCF sensitivity analysis. Discontinuation occurred in 70% of the overall population. Approximately 74% of triptan users purchased only one type of triptan formulation, 19% purchased two types of triptan formulations, and 6.8% purchased three or more.

While the median treatment time among patients who discontinued triptan use (n = 28,920, 70%) was 3 months (IQR: 3 months to 1.3 years), the median treatment time among patients who did not discontinue (n = 12,377, 30%) was 2.5 years (IQR: 9.3 months to 6.1 years). After excluding those with only a single dispensation, 12,185 out of the remaining 21,393 individuals (56.9%) discontinued use, with an overall median treatment time of 4.6 years (95% CI: 4.5–4.7 years, see Figure 2B).

**Figure 3:**
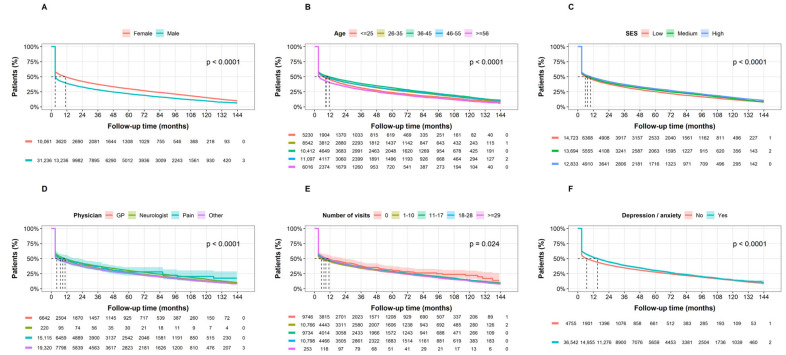
Kaplan-Meier curves of triptan treatment discontinuation by patient characteristics. Time to treatment discontinuation (months) from triptan initiation stratified by: (**A**) sex; (**B**) age at initiation (≤25, 26–35, 36–45, 46–55, ≥56 years); (**C**) socioeconomic status (low, medium, high); (**D**) prescribing physician specialty (general practitioner, neurologist, pain specialist, other); (**E**) healthcare utilization in the prior year (0, 1–10, 11–17, 18–28, ≥29 visits); and (**F**) prevalent anxiety and/or depression in the prior year. Groups were compared using the log-rank test. Numbers below curves represent individuals at risk at each time point. Dashed lines mark the median time of each curve. SES: socioeconomic status, GP: general practitioner.

In subgroup analyses by sex (Figure 3A), by age at treatment initiation (Figure 3B), by SES (Figure 3C), by first prescribing physician (Figure 3D), and by number of visits to any health provider during the year prior treatment initiation (Figure 3E), the median time to discontinuation was always under 12 months. Only individuals with anxiety and/or depression diagnosis during the year prior to treatment initiation had slightly higher median time to discontinuation (Figure 3F).

**Figure 4:**
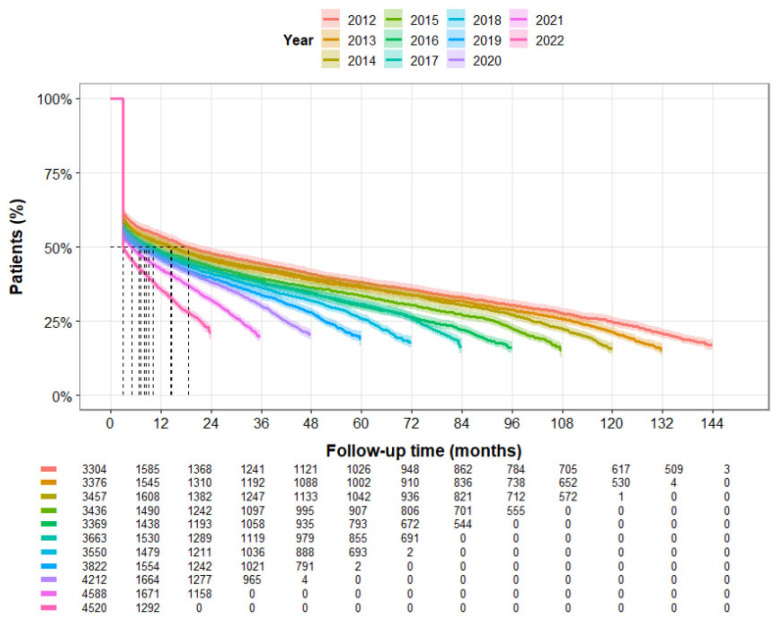
Kaplan-Meier curves of triptan treatment discontinuation by year of treatment initiation. Time to triptan discontinuation stratified by year of treatment initiation (2012–2022, each year shown separately). Numbers represent individuals at risk at each time point. Dashed lines mark the median time of each curve.

When assessing annual discontinuation rate, the median time to discontinuation remains under 12 months almost for all calendar years throughout the study period (Figure 4). Interestingly, it showed a consistent yearly decrease, which was significant for the entire study period.

### Medical Censorship

Of 41,297 individuals with at least one dispensation, censorship occurred among 12,377 (30.0%) members. While 5940 individuals (14.4%) were censored due to contraindications related to triptans, 6397 individuals (15.5%) had an administrative censorship at the end of follow-up. The most common contraindication was the initiation of SSRI therapy, affecting 3312 individuals (8%). Other reasons for censorship included incident pregnancy (5.8%) and IHD (0.4%).

## 4. Discussion

In this large, population-based retrospective cohort study spanning over a decade, we identified a concerning pattern of triptan use, suggesting a significant undertreatment of acute migraine in Israel. Specifically, we observed: (1) a much lower-than-expected proportion of treated patients; (2) a relatively late age of triptan initiation; and (3) short treatment persistence with high discontinuation rates. Only 3.4% (91,619 triptan users during study period out of 2.8 million MHS members) had used triptans for acute migraine treatment. In contrast, previous retrospective cohort studies in Israel estimated migraine prevalence at 5.2% [21] and 7.65% [22], though these figures likely also underestimate the true prevalence due to reliance on retrospective diagnoses and/or prescriptions. Given the ethnic diversity of Israeli society, there is no reason to assume migraine prevalence differs substantially from global estimates, which range from 9.7 to 16.4% [26] or even higher [1]. If even half of Israeli migraine patients were expected to use triptans, our findings indicate strikingly low utilization.

This is particularly noteworthy considering the accessibility of professional medical care in Israel, the internationally recognized quality monitoring in primary care [27], and the high standard of healthcare overall [28]. Since triptans are recommended as first-line treatment for acute migraine, the observed underuse may reflect a broader issue in migraine management. Although we did not directly investigate reasons for low triptan usage, possible explanations include perceptions of triptans as “strong” medications; concerns about adverse effects; underestimation of migraine as a serious medical condition; and a reactive rather than proactive approach to treatment by physicians.

The mean age at triptan initiation was approximately 38 years. While this late onset aligns with findings from existing literature [11,22,29], considering that migraine typically manifests during childhood or adolescence, it underscores a substantial delay in initiating appropriate treatment. Although our study was not designed to identify the causes of this delay, potential contributing factors may include patient beliefs regarding triptans and prescription medications versus over-the-counter drugs, or deliberate treatment postponement during reproductive years. We hypothesize that a more proactive approach by treating physicians could help bridge this gap and improve the timeliness and effectiveness of migraine management.

Approximately 48% of the study population discontinued triptan use after a single dispensation, and 70% discontinued treatment during the overall study period. It is important to note that these patients were not lost to follow-up in the traditional sense; rather, they remained within the healthcare system but ceased triptan therapy. These results support previous findings across the US, Europe, and Asia [10,11,13,16,23,29,30], confirming that the high rate of ‘one-and-done’ users is a global phenomenon.

This early ‘drop-off’ suggests a critical period of treatment vulnerability within the first 90 days of initiation. The impact of such high initial discontinuation is twofold: first, it indicates that nearly half of patients may experience immediate dissatisfaction due to perceived lack of efficacy or side effects; second, it reflects a missed opportunity for clinical intervention. Given that 75% of patients used only one type of triptan, the majority of those who discontinued did so without attempting a second agent, despite clinical guidelines recommending trials of multiple triptans to identify the most effective individual response. This suggests that the ‘first-year drop’ is a major driver of chronic migraine undertreatment in the real-world setting.

Switching rates among different triptans in the current study were low but appear slightly higher than those reported in the literature [10], with only about 5–10% of patients trying more than one triptan after their initial prescription. Most patients who discontinued their first triptan did not switch to another triptan but instead stopped prescription therapy or reverted to non-specific acute medications such as NSAIDs or opioids [10,11]. These discontinuation patterns are unlikely to be explained by the natural remission of migraine symptoms observed in specific populations, such as postmenopausal women. Rather, they are probably more commonly associated with lack of efficacy, and side effects. Other factors linked to discontinuation include higher migraine-related disability, comorbid depression, opioid use, and prescriptions by non-specialists [31].

We noted slightly better adherence to triptan treatment when it was prescribed by a primary care physician compared to a neurologist or pain specialist. Sex, ethnicity, and area of residence did not influence the results. Similarly, there was no change in the pattern of triptan usage during the coronavirus pandemic or following the market introduction of gepants. As mentioned, a consistent yearly decrease in the median time interval for discontinuation was observed throughout the study period. While the emergence and integration of new migraine treatments, specifically CGRP monoclonal antibodies and gepants from 2018 onwards, may provide a partial explanation for these shifting trends in later years, the cause for it in earlier years remains speculative. As we did not formally assess the impact of these specific market entries, further investigation into other large-scale cohorts is warranted to determine the underlying causes of this increasing rate of discontinuation. Although not being an absolute contraindication for triptan use, SSRI use emerged as the main reason for medically “discontinuing” triptan in our study. This practice likely stems from concerns about the potential risk of serotonin syndrome when triptans are used concomitantly with SSRIs or selective norepinephrine reuptake inhibitors (SNRIs), following a 2006 FDA advisory warning which was retracted sometime after. Subsequent studies and reviews have demonstrated that the actual incidence of serotonin syndrome in patients using both triptans and SSRIs/SNRIs is very low [32]. The American Headache Society’s position paper concluded that available evidence does not support limiting the use of triptans with SSRIs or SNRIs due to concerns for serotonin syndrome, though they recommend vigilance for symptoms of serotonin toxicity [33].

The generalizability of our findings should be considered within the context of Israel’s universal healthcare system. In MHS, triptans are heavily subsidized and accessible through community-based clinics, which minimizes the impact of cost as a barrier to persistence. Consequently, the high rate of discontinuation observed here likely reflects clinical dissatisfaction (lack of efficacy or side effects) rather than financial constraints. In healthcare systems without universal coverage or with higher out-of-pocket costs, it is probable that discontinuation rates would be even higher due to the compounding effect of economic barriers.

Our study has several limitations. Firstly, consistent with other real-world evidence, we were unable to identify individuals with migraine due to under- and mis-documentation [34]. This may lead to a “healthy user” bias, where our cohort represents patients with more frequent or severe attacks who actively seek medical care, potentially overestimating the global burden of triptan discontinuation. Secondly, our database lacks information on migraine features such as frequency, intensity, additional symptoms, or reasons for discontinuation. Consequently, we could not distinguish between discontinuation due to lack of efficacy versus discontinuation due to treatment success or clinical remission, though the high rates of early cessation suggest the former. Thirdly, we did not assess whether patients initiated preventive therapy as a reason for triptan discontinuation, although previous research by Thomsen et al. has shown that low triptan adherence cannot be explained by successful prophylactic treatment [35]. However, the lack of these data prevents us from exploring the transition from acute to preventive care as a potential driver of switching patterns. Fourth, we did not assess triptan overdose. This limitation means that while we highlight undertreatment, a subset of the population may simultaneously be experiencing mismanagement through over-utilization, a nuance that administrative dispensation data cannot fully capture.

## 5. Conclusions

Our findings reveal lower than expected utilization of triptans among individuals suffering from migraine in Israel, in addition to global trends of significant acute migraine undertreatment, characterized by poor persistence and adherence to triptan therapy, the established standard of care. This pattern suggests that a substantial portion of individuals, in their most productive years, experience unnecessary reductions in quality of life and, in some cases, disability. Our results suggest that the current “standard of care” often results in a “one-and-done” approach, where patients abandon therapy before exploring the full range of available triptan formulations. Clinicians should anticipate this early vulnerability by scheduling a follow-up visit or communication within the first 90 days of the initial prescription.

## Figures and Tables

**Table 1:** Baseline characteristics.

	Study Cohort N = 41,297
**Age at first triptan (years)**	
Mean (SD)	39.8 (13.3)
Median (IQR)	38.6 (28.4, 48.1)
Range	21.0–100.1
≤25, *n* (%)	6016 (14.6%)
26–35, *n* (%)	11,097 (26.9%)
36–45, *n* (%)	10,412 (25.2%)
46–55, *n* (%)	8542 (20.7%)
≥56, *n* (%)	5230 (12.7%)
**Sex**	
Female, *n* (%)	31,236 (75.6%)
Male, *n* (%)	10,061 (24.4%)
**Socio-economic status**	
High, *n* (%)	14,723 (35.7%)
Medium, *n* (%)	13,694 (33.2%)
Low, *n* (%)	12,833 (31.1%)
Missing, *n* (%)	47 (0.1%)
**Resident area**	
North, *n* (%)	7874 (19.1%)
Center, *n* (%)	26,843 (65.0%)
South, *n* (%)	6580 (15.9%)
**Smoking status**	
Ever, *n* (%)	14,366 (34.8%)
Never, *n* (%)	26,459 (64.1%)
Missing, *n* (%)	472 (1.1%)
**Socio-religious affiliation**	
General Jewish population, *n* (%)	36,389 (88.1%)
Arab faith, *n* (%)	3198 (7.7%)
Ultra-orthodox Jew, *n* (%)	1710 (4.1%)
**≥1 HRT/contraceptives dispensation**	
1-year prior index date, *n* (%)	7678 (18.6%)
Ever prior index date, *n* (%)	18,939 (45.9%)
**Anxiety and/or depression diagnosis**	
1-year prior index date, *n* (%)	4755 (11.5%)
Ever prior index date, *n* (%)	15,555 (37.7%)
**Irritable bowel syndrome diagnosis**	
1-year prior index date, *n* (%)	568 (1.4%)
Ever prior index date, *n* (%)	3317 (8.0%)
**Cervical pain diagnosis**	
1-year prior index date, *n* (%)	3790 (9.2%)
Ever prior index date, *n* (%)	14,740 (35.7%)
**Fibromyalgia diagnosis**	
1-year prior index date, *n* (%)	678 (1.6%)
Ever prior index date, *n* (%)	1486 (3.6%)
**Fatigue diagnosis**	
1-year prior index date, *n* (%)	3351 (8.1%)
Ever prior index date, *n* (%)	17,454 (42.3%)
**Insomnia diagnosis**	
1-year prior index date, *n* (%)	1468 (3.6%)
Ever prior index date, *n* (%)	6263 (15.2%)
**Osteoarthritis diagnosis**	
1-year prior index date, *n* (%)	790 (1.9%)
Ever prior index date, *n* (%)	2803 (6.8%)
**Rheumatoid arthritis diagnosis**	
1-year prior index date, *n* (%)	120 (0.3%)
Ever prior index date, *n* (%)	446 (1.1%)
**Lupus diagnosis**	
1-year prior index date, *n* (%)	64 (0.2%)
Ever prior index date, *n* (%)	144 (0.3%)
**Gout diagnosis**	
1-year prior index date, *n* (%)	90 (0.2%)
Ever prior index date, *n* (%)	436 (1.1%)
**Rheumatic disease diagnosis**	
1-year prior index date, *n* (%)	1032 (2.5%)
Ever prior index date, *n* (%)	3510 (8.5%)
**Overactive bladder diagnosis**	
1-year prior index date, *n* (%)	229 (0.6%)
Ever prior index date, *n* (%)	968 (2.3%)
**First prescribing physician**	
General physician, *n* (%)	19,320 (46.8%)
Neurologist, *n* (%)	15,115 (36.6%)
Pain, *n* (%)	220 (0.5%)
Other specialty, *n* (%)	6642 (16.1%)
**Treatment initiation (calendar years)**	
2012, *n* (%)	3304 (8.0%)
2013, *n* (%)	3376 (8.2%)
2014, *n* (%)	3457 (8.4%)
2015, *n* (%)	3436 (8.3%)
2016, *n* (%)	3369 (8.2%)
2017, *n* (%)	3663 (8.9%)
2018, *n* (%)	3550 (8.6%)
2019, *n* (%)	3822 (9.3%)
2020, *n* (%)	4212 (10.2%)
2021, *n* (%)	4588 (11.1%)
2022, *n* (%)	4520 (10.9%)
**Number of visits to any health provider—1 year prior**	
0, *n* (%)	253 (0.6%)
1–10, *n* (%)	10,798 (26.1%)
11–17, *n* (%)	9734 (23.6%)
18–28, *n* (%)	10,766 (26.1%)
≥29, *n* (%)	9746 (23.6%)

HRT: Hormone replacement therapy; SD: standard deviation, IQR: interquartile range.

## Data Availability

The study data included individual-level sensitive information. According to the regulation of the Israeli Ministry of Health (01/18) and MHS’s data privacy policy, patient-level data (including de-identified information) cannot be transferred outside MHS’s premise. Queries regarding the data can be addressed to: barer_y@mac.org.il.

## Abbreviations

The following abbreviations are used in this manuscript:

ATC	Anatomical Therapeutic Chemical
CI	Confidence interval
CPT	Current Procedural Terminology
GP	General practitioner
HRT	Hormone replacement therapies
IBS	Irritable bowel syndrome
ICD-9-CM	International Classification of Diseases, Ninth Revision, Clinical Modification
IHD	Ischemic heart disease
IQR	Interquartile range
LOCF	Last observation carried forward
MAOI	Monoamine oxidase inhibitor
MHS	Maccabi Healthcare Services
OAB	Overactive bladder syndrome
SD	Standard Deviation
SES	Socioeconomic status
SSRI	Selective Serotonin Reuptake Inhibitor
TIA	Transient ischemic attack
